# Design, Synthesis and Antihyperglycemic/Antilipidemic Activities of Quinoline-thiazolidine-2,4-dione Hybrid

**DOI:** 10.3390/ph19091490

**Published:** 2026-09-19

**Authors:** ElShimaa Ahmed Mohamed Abousaada, Asmaa Galal-Khallaf, Khaled Mohammed-Geba, Mohamed El-Bahnsawye, Mona K. Abo Hussein, Elshaymaa I. Elmongy, Rania Ali Ahmed Shaltout, Reem Binsuwaidan, Hadeer Ali, Ahmed I. El-Tantawy, Hamed Abdel-Bary, Ibrahim El Tantawy El Sayed

**Affiliations:** 1 Chemistry Department, Faculty of Science, Menoufia University, Shebin El-Kom 32511, Egypt; alshaimaaahmed95@gmail.com (E.A.M.A.); m_nh22010@yahoo.com (M.E.-B.); yohader1@gmail.com (H.A.); hamedabdelbary2006@yahoo.com (H.A.-B.); ibrahimtantawy@science.menofia.edu.eg (I.E.T.E.S.); 2 Zoology Department, Molecular Biology and Biotechnology Laboratory, Faculty of Science, Menoufia University, Shebin El-Kom 32511, Egypt; as_kh_22@yahoo.com (A.G.-K.); khaledmohammed@science.menofia.edu.eg (K.M.-G.); raniashaltout606@gmail.com (R.A.A.S.); 3 Clinical Microbiology and Immunology Department, National Liver Institute, Menoufia University, Shebin El-Kom 32511, Egypt; monaabohussein@yahoo.com; 4 Pharmaceutical Chemistry Department, Faculty of Pharmacy, Capital University (Formerly Helwan University), Ain Helwan, Cairo 11795, Egypt; 5 Department of Pharmaceutical Sciences, College of Pharmacy, Princess Nourah Bint Abdulrahman University, P.O. Box 84428, Riyadh 11671, Saudi Arabia; rabinsuwaidan@pnu.edu.sa; 6 Research Institute for Interdisciplinary Science, Okayama University, Tsushima-naka, Kita-ku, Okayama 700-8530, Japan; chemahmed293@gmail.com

**Keywords:** quinoline, thiazolidinedione, diabetes, zebrafish, hyperglycemic, hybrid, hyperlipidemic

## Abstract

**Background:** Type 2 diabetes mellitus (T2DM) and hyperlipidemia are interrelated metabolic disorders that significantly increase cardiovascular risk. Thiazolidinediones are known insulin sensitizers, whereas quinoline derivatives possess diverse pharmacological activities. To explore their complementary activity, we designed a novel hybrid compound integrating a thiazolidinedione core with a quinoline moiety via a carbon spacer. **Materials and Methods:** The hybrid molecule was synthesized through a straightforward route and structurally confirmed by spectroscopic analyses. Zebrafish (Danio rerio) were employed as an in vivo model, with hyperglycemia induced by immersion in 100 mM glucose monohydrate for 12 days. Fish were divided into treatment groups receiving 20 or 80 mg/kg of the compound, alongside the controls. Biochemical parameters (glucose, triglycerides, insulin) and gene expression levels (NF-κB, ACOX1, ACCα) were assessed. In silico docking was conducted to predict molecular interactions. **Results:** Treatment with the hybrid compound significantly restored glucose, triglyceride, and insulin levels. High-dose administration suppressed NF-κB expression and normalized ACOX1 levels, while both low- and high-dose treatments downregulated ACCα compared with untreated hyperglycemic controls. Computational studies supported multitarget activity through predicted binding to PPAR-α and ACOX1. **Conclusions:** Quinoline-Thiazolidine-2,4-Dione demonstrated strong antihyperglycemic and antihypertriglyceridemic activities in zebrafish models, supported by in silico validation.

## 1. Introduction

Diabetes mellitus (DM), commonly referred to as diabetes, is a chronic detrimental condition characterized by consistently elevated blood glucose levels. This condition arises from either insufficient insulin production or the inability of the body to use insulin effectively. Diabetes affects individuals across all demographics, including age, sex, and geographic location, and is a leading cause of global mortality and morbidity. The development of type 2 diabetes, which constitutes over 90% of all cases, is influenced by both genetic and environmental factors [1]. Diabetes was first recorded in an Egyptian manuscript dating back approximately 3000 years [2]. The World Health Organization (WHO) defines diabetes mellitus as a chronic metabolic disorder characterized by elevated blood glucose levels. This condition can progressively damage the heart, blood vessels, kidneys, eyes, and nerves [3]. In 2025, the International Diabetes Federation (IDF) reported that approximately 589 million individuals aged 20 to 79 years were affected by diabetes, representing 11.1% of all adults within this age range. Projections indicate that by 2050, the number of adults with diabetes will increase to 852.5 million. Although the global population is expected to grow by 25% over the next 25 years, the prevalence of diabetes is anticipated to rise by 45%. The Middle East and North Africa region, with 85 million individuals affected by diabetes, ranks as the third most impacted region globally by type 2 diabetes mellitus (T2DM), following the Western Pacific and Southeast Asia [4]. Gestational diabetes and type 1 diabetes are the other two forms of diabetes [5,6]. As the condition progresses, insulin secretion becomes insufficient to maintain glucose homeostasis, leading to hyperglycemia. Patients with type 2 diabetes mellitus (T2DM) are primarily characterized by obesity or an increased body fat percentage, predominantly located in the abdominal region. The global increase in obesity, sedentary lifestyles, high-calorie diets, and an aging population are the primary drivers of the T2DM epidemic, with the incidence and prevalence of the disease having quadrupled [7]. The incidence and prevalence of T2DM vary by geographical region, with over 80% of patients residing in low to middle-income countries, complicating treatment efforts. Obesity induces insulin resistance, which is evident in the liver, white adipose tissue, and skeletal muscle. Consequently, pancreatic beta cells secrete insufficient insulin to counteract this resistance, a phenomenon observed as an early global challenge [8,9].

Several pharmacological agents enhance cellular responsiveness to insulin and function as insulin sensitizers in patients with DM. Notably, thiazolidinediones (TZDs) enhance insulin activity and sensitivity in essential organs by modulating intracellular metabolic pathways [10]. Thiazolidine-2,4-dione (TZD) is a heterocyclic compound with diverse applications in medicinal chemistry. Compounds with the thiazolidine-2,4-dione nucleus have demonstrated promising activity in various categories, including anti-hyperglycemic, aldose reductase inhibitors, anti-inflammatory, anti-cancer, anti-arthritic, and anti-microbial activities. Consequently, these compounds are essential building blocks for developing novel therapeutic agents. A broad range of biological activities has been made possible by different substituents on the thiazolidine-2,4-dione nucleus [11]. TZDs also enhance insulin-dependent glucose uptake in muscle and adipose tissues, reduce hepatic gluconeogenesis, and elevate adiponectin levels. Adiponectin, a cytokine secreted by adipose tissue, promotes fatty acid oxidation and improves insulin sensitivity when administered in conjunction with TZD therapy [12,13,14].

TZDs regulate the expression of specific genes by binding to the nuclear transcription regulator peroxisome proliferator-activated receptor-gamma (PPAR-gamma) [13]. Upon activation of PPARs by their ligands, control of glucose metabolism, fatty acid storage, and adipocyte differentiation occurs in the muscle, fat, and liver [15]. The binding of thiazolidinediones (TZDs) induces conformational changes that alter the expression of various genes associated with metabolic regulation, including fatty acyl-CoA synthase, glucokinase, and lipoprotein lipase [16]. PPAR-gamma agonists enhance adiponectin and GLUT4 expression while mitigating the impact of TNF-alpha on adipocytes, thereby decreasing insulin resistance. The upregulation of GLUT4 facilitates improved glucose uptake by both adipocytes and skeletal muscle cells in response to insulin [15]. This class of medications includes englitazone, pioglitazone, ciglitazone, troglitazone, and rosiglitazone. These medications have strong anti-diabetic effects. However, some have unfavorable side effects, such as genotoxicity and hepatotoxicity [17,18]. Therefore, the development of novel thiazolidinedione analogs with minimal toxicity and equivalent or superior therapeutic benefits is necessary. Quinoline-thiazolidinedione hybrids have been reported to have synergistic therapeutic potential as anti-diabetic agents [19]. The molecular hybridization technique (MHT) is a key approach for developing novel hybrid analogs with improved pharmacological profiles, especially as antidiabetic agents. It combines distinct pharmacophoric moieties within one molecule to enhance efficacy, selectivity, and multitarget potential. In this study, thiazolidinedione (TZD) and quinoline scaffolds were hybridized to synthesize a quinoline–thiazolidinedione hybrid, evaluate its dual antidiabetic/hypolipidemic efficacy in zebrafish models using biochemical and gene-expression protocols, and validate its molecular interactions through docking studies with PPAR-α and ACOX1. This hybridization strategy aims to optimize the chances of finding a molecule that effectively interacts with the target, leading to the desired therapeutic effects.

## 2. Results

### 2.1. Synthesis and Elucidation of Hybrid

**Scheme 1:**
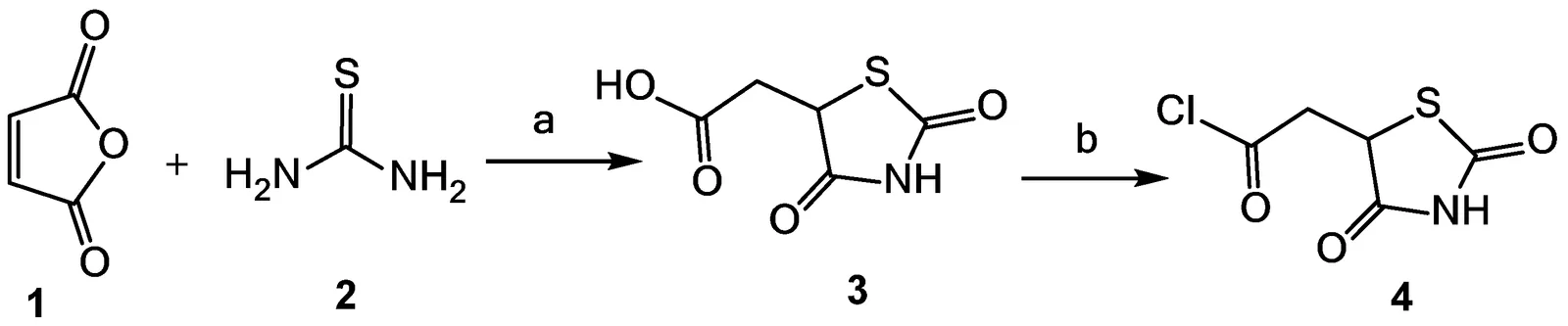
Synthesis of 2-(2,4-dioxothiazolidin-5-yl) acetyl chloride **4**. Reagents and conditions: (a) Conc HCl. reflux, 5h. (b) SOCl_2_ in dioxane, reflux, 5 h.

The 2, 4-Thiazolidinedione-5-acetic acid **3** [20] was prepared in good yield from the reaction of maleic anhydride **1** with thiourea **2** in the presence of concentrated hydrochloric acid. Further reaction of **3** with SOCl_2_ in anhydrous dioxane afforded the corresponding acid chloride **4** in good yield [20], Scheme 1.

**Scheme 2:**
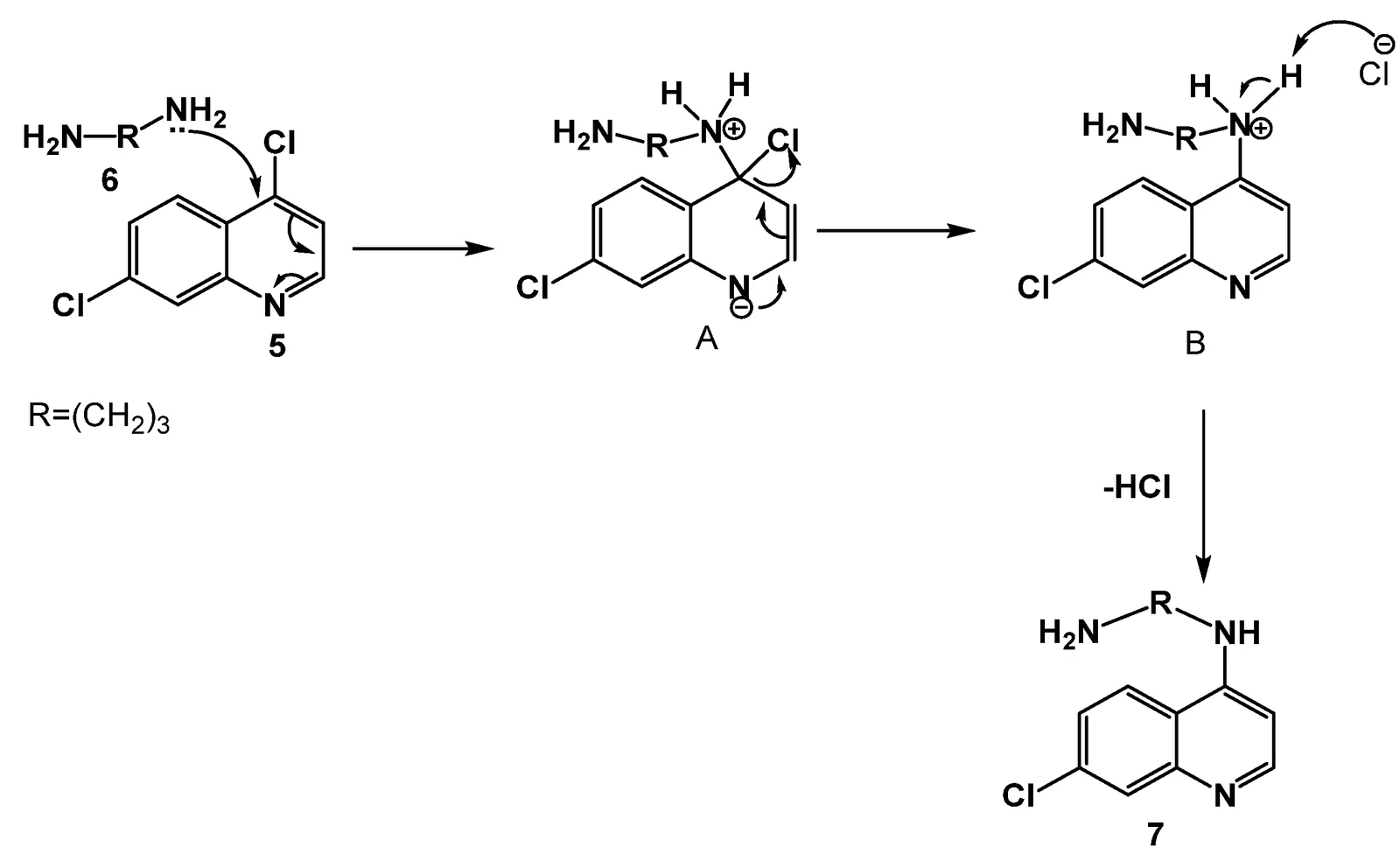
Suggested mechanism for the synthesis of aminopropylaminoquinoline **7**.

The refluxing of 4,7-dichloroquinoline **5** with 1,3-diaminopropane **6** under neat conditions for 2 h afforded N′(7-chloroquinolin-4-yl) propane-1,3-diamine **7** as the intermediate product after work-up, with a melting point of 180–200 °C. The reaction proceeds via nucleophilic substitution of the amino group of 1,3-diaminopropane by chlorine at the C-4 position of 4,7-dichloroquinoline, affording intermediate **7** as a yellow product in good yield. N′ (7-chloroquinolin-4-yl) propane-1,3-diamine **7** was coupled with 2,4-dioxothiazolidine-5-acetyl chloride 4 in the presence of triethylamine as the base in DMF under refluxing conditions for 6 h until completion of the reaction, as monitored by TLC. The plausible mechanism for the formation of **7** likely proceeds via nucleophilic aromatic substitution (SNAr), in which the nucleophilic attack of the amine group on the unsaturated C-4-chlorine-bearing **5** is followed by the release of chloride. The nucleophilic aromatic substitution of the chloride atom at the 4th position of the quinoline ring core by bis-amine **6** proceeds through the addition of the amino group (: Nu–) to form a resonance-stabilized anion with a new C– N bond, followed by the elimination of HCl to afford the final product **7,** as shown in Scheme 2.

The IR spectra of intermediates **7** showed an absorption band ranging from υ: 3437 to 3212 cm^−1^, which represents the (NH and NH_2_) groups, respectively. Moreover, the ^1^H-NMR spectrum showed characteristic exchangeable peaks ranging from δ = 9.51 to 12.44 ppm, which are characteristic of (NH_2_ and NH) groups. For mass spectroscopy, the presence of the expected molecular ion peaks in the synthesized structures confirmed the formation of the desired compounds. The presence of one chloride was confirmed by mass spectrometry, with an M^+2^ ion peak at a 4:1 height ratio to the M^+^ peak. 

**Scheme 3:**
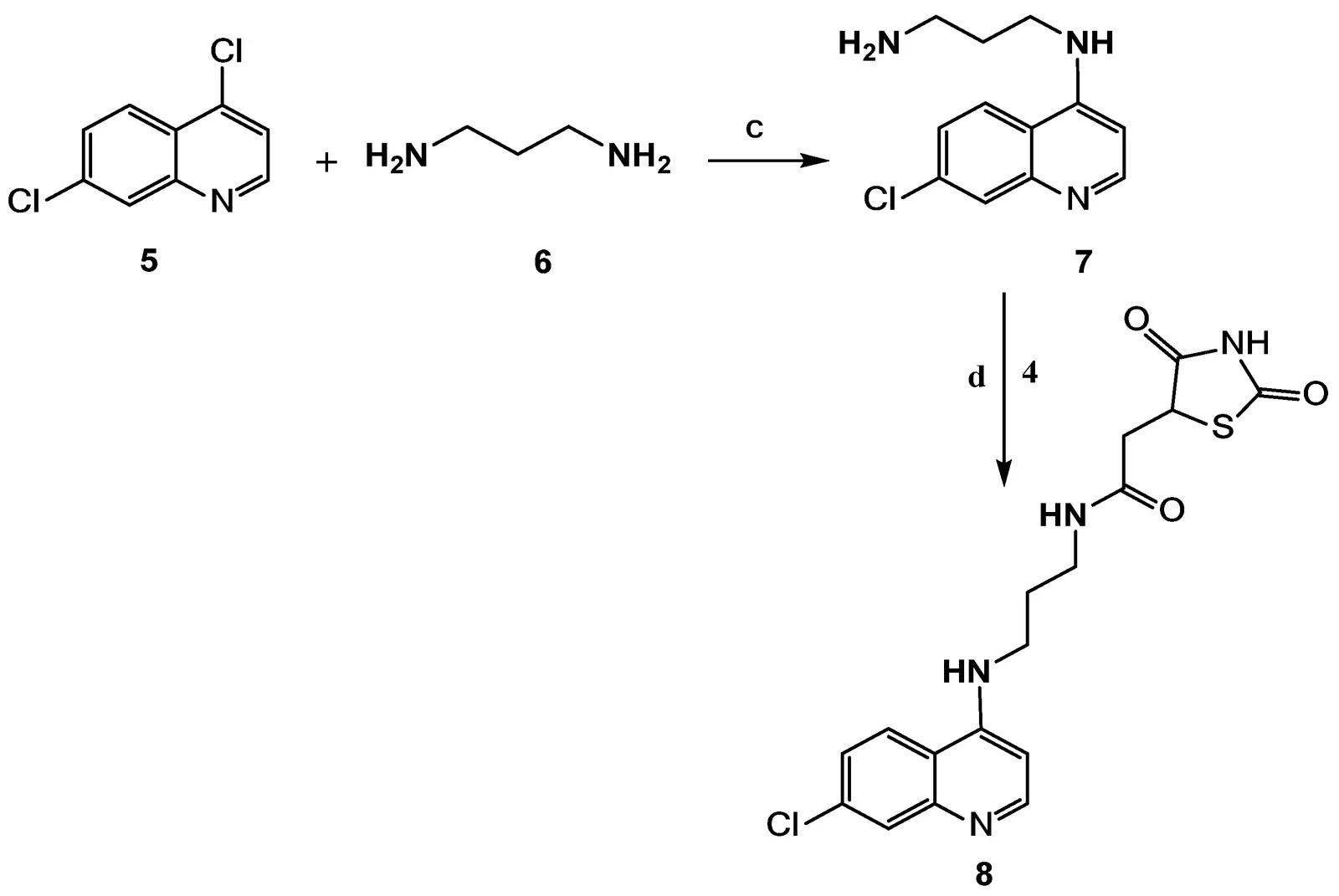
Synthesis of quinoline-thiazolidine-2,4-dione hybrid **8**. Reagents and conditions: (c) 5eq.1,3-diaminopropane 6, reflux, 2 h. (d) dry DMF, TEA (3eq.), reflux, 6 h.

Finally, the new hybrid of the quinoline-thiazolidinedione scaffold **8** was synthesized by reacting 4-aminoalkylaminoquinoline intermediates **7** with 2, 4-thiazolidinedione-5-acid chloride **4**, as shown in Scheme 3. The newly synthesized hybrid **8** was confirmed by FTIR, ^1^H NMR, ^13^C NMR, and ESI-MS analyses. The FTIR spectrum recorded in ATR mode showed characteristic absorptions at 3410 and 3245 cm^−1^, attributable to N–H stretching vibrations of the thiazolidinedione and amide functionalities, respectively. The strong absorptions at 1735 and 1685 cm^−1^ were assigned to the carbonyl groups of the thiazolidinedione and amide moieties, with partial overlap of carbonyl absorptions. The bands at 1590 and 1535 cm^−1^ were consistent with the quinoline C=C and C=N vibrations, respectively, while the absorption at 725 cm^−1^ supported the presence of the C–Cl bond. The ^1^H NMR spectrum (500 MHz, DMSO-d_6_) further supported the proposed structure. The downfield singlets at δ 10.22 and 9.10 ppm were assigned to the thiazolidinedione NH and quinoline-linked NH protons, respectively, whereas the singlet at δ 8.50 ppm was attributed to the amide NH. Moreover, the characteristic CH of the thiazolidinedione ring signal was assigned at δ 4.65 ppm. Furthermore, the methylene group of propyl spacer showed at δ 3.30 and 2.90 ppm, and the methylene group adjacent to the thiazolidinedione ring was recorded at δ 3.10 ppm. The methylene signal at δ 1.85 ppm was consistent with the internal CH_2_ group of the propyl chain. The methylene group adjacent to NH resonated at δ 3.40–3.30 ppm, while the diastereotopic methylene protons of the thiazolidinedione side chain appeared as two doublets of doublets at δ 3.10 and 2.90 ppm. the presence of two doublets of doublets at ~2.90–3.10 ppm and~ 3.10–4.62 ppm is due to the presence of two diasterotopic protons corresponding to the exocyclic methylene group directly attached to the thiazolidinone ring. These two protons displayed coupling constants ranging from JAB = 15.21–16.04 Hz, –CHHb-JAX = 6.5–10.51 Hz, and JBX = 3.9–6.7 Hz, respectively. The high value of JAB agrees with the data reported by Takahashi owing to the carbonyl effect’) for structurally related 2-thioxo-4-thiazolidinone-acetic acids [21]. The ^13^C NMR spectrum (125 MHz, DMSO-d_6_) displayed resonances attributable to the carbonyl, heteroaromatic, and aliphatic carbons of hybrid 8. The signals at δ 196.01 and 167.50 ppm were assigned to the two nonequivalent carbonyl carbons of the thiazolidinedione ring, whereas the resonance at δ 171.13 ppm corresponded to the amide carbonyl. The quinoline carbons resonated over the δ 152.41–122.56 ppm range. The aliphatic region contained signals at δ 55.20 and 42.03 ppm, assigned to the thiazolidinedione C-5 and CH_2_–NH groups respectively, together with the methylene resonance at δ 29.52 ppm. The molecular ion peaks observed in the mass spectra were fully consistent with the calculated molecular formulas of new synthesized compounds 8. Eventually, the purity of synthesized conjugate **8** was evaluated using HPLC showing that the compound is sufficiently pure (95%) for further biological evaluation, Supplementary Material File S1.

### 2.2. Interpretation of Docking Study

**Figure 1:**
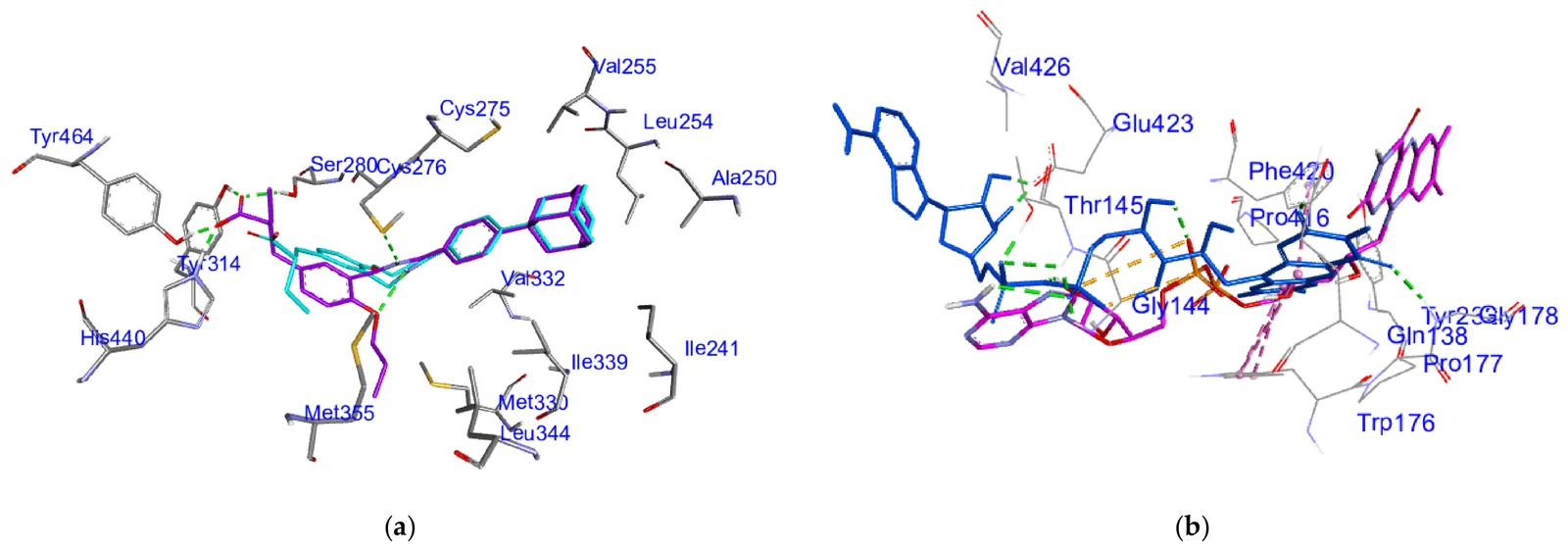
(**a**) Redocking of the co-crystalized ligand of PPAR-α for validation with RMSD deviation (1.61 Å); where the co-crystallized ligand is represented by magenta sticks and the redocked one is represented in cyan sticks; H-bonds are present in green dashed lines. (**b**) Redocking of the co-crystalized ligand of ACOX-1 for validation with RMSD deviation (1.62 Å); where the co-crystallized ligand is represented by blue sticks and the redocked one is represented in pink sticks; H-bonds are present in green dashed lines.

**Figure 2:**
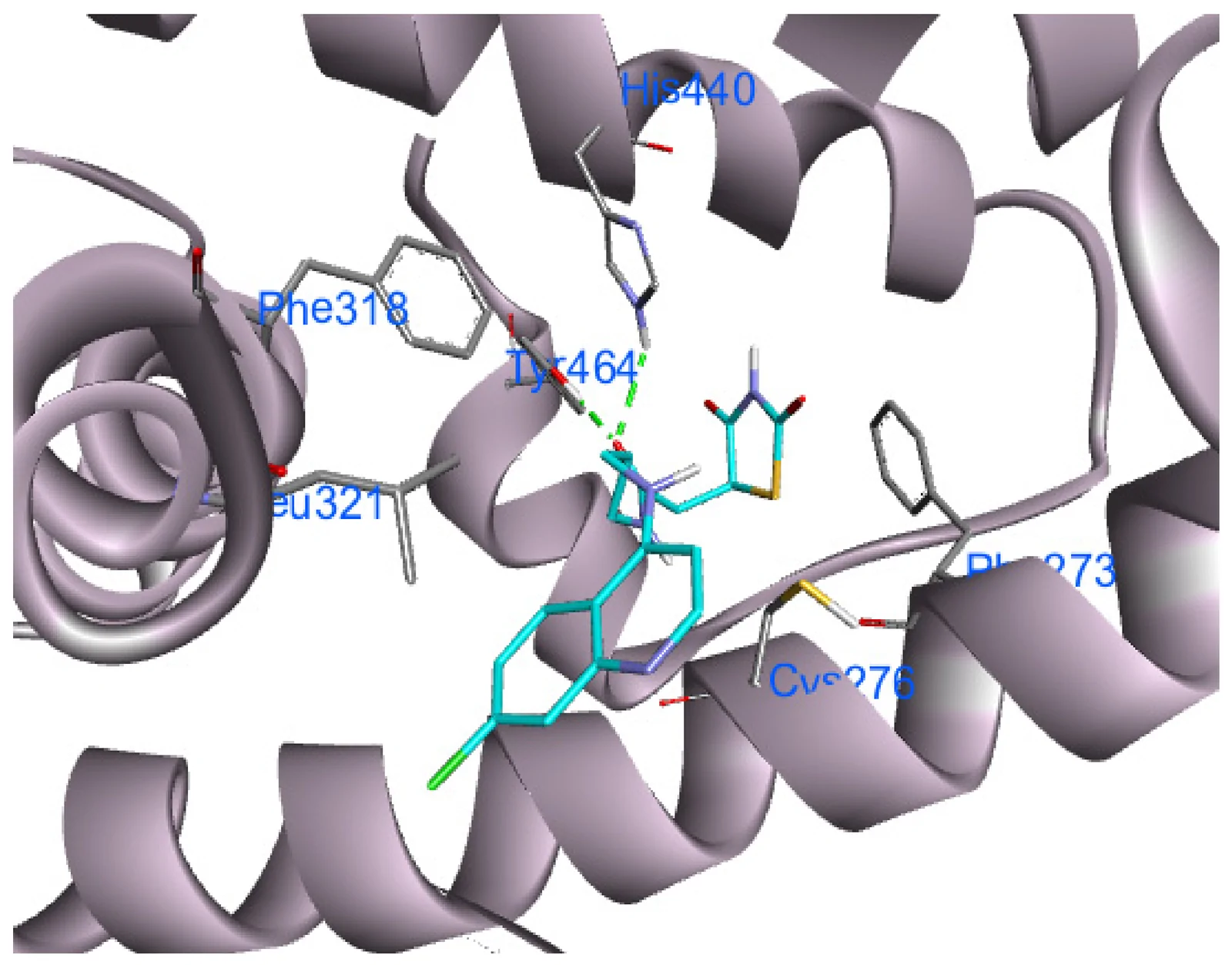
3D-interactions of Quinoline-Thiazolidine-2, 4-Dione **8** (in cyan-colored sticks) in PPAR-α binding site where H-bonds are represented by green dashed lines.

**Figure 3:**
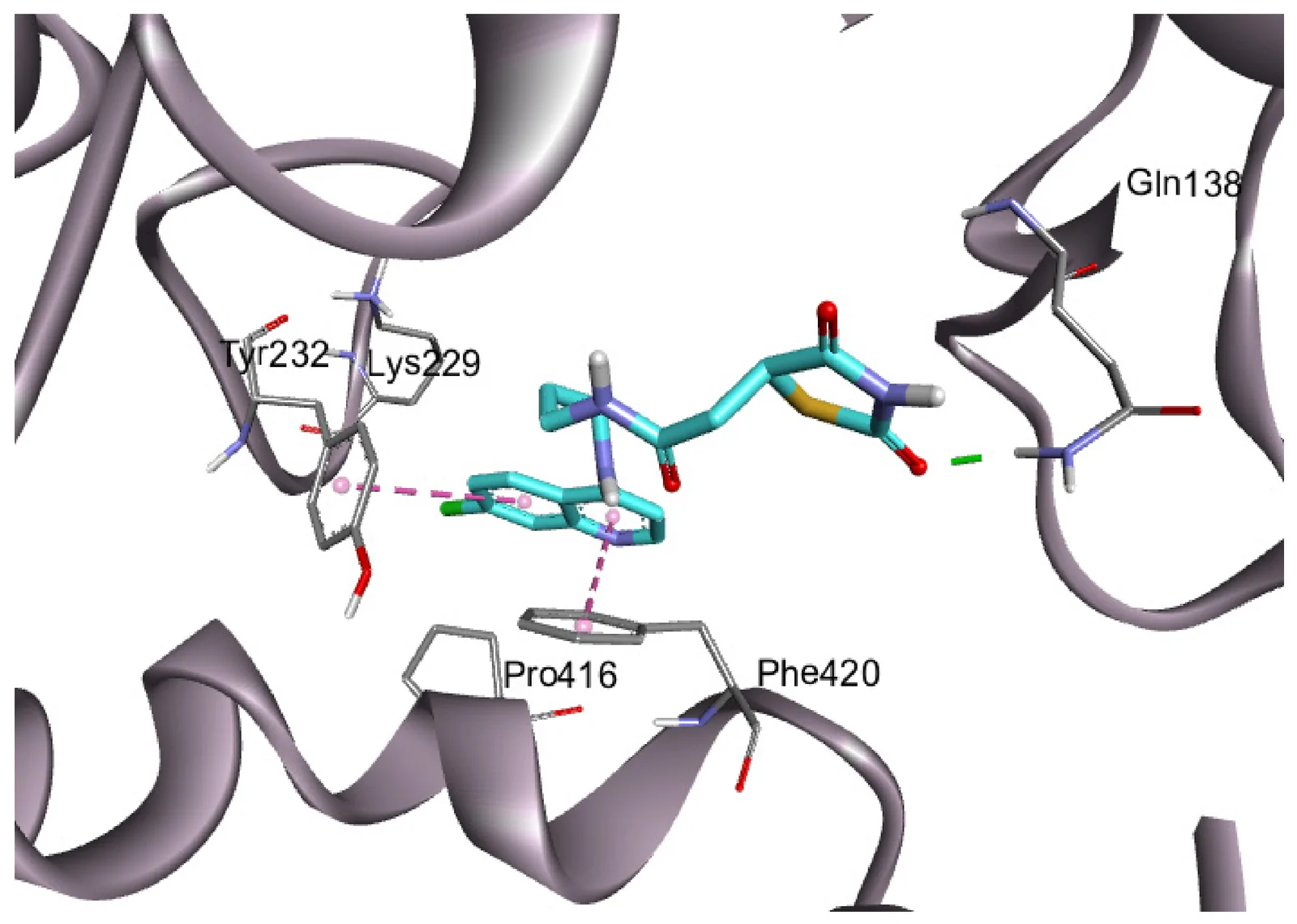
Residues of interactions between Quinoline-Thiazolidine-2, 4-Dione **8** represented in blue sticks and the binding pocket of the target protein ACOX-1, hydrogen bonds are in green dashed lines, and hydrophobic interactions are in magenta dashed lines.

The synthesized Quinoline-Thiazolidine-2, 4-Dione **8** was docked into the ligand-binding cavities of PPAR-α (PDB: 2ZNN) and ACOX-1 (PDB: 7Q86). The compound has a chiral center so both enantiomers were investigated for the target proteins and the best scores were recorded. Both enantiomers had comparable results against PPARα, however the R-enantiomer showed better results against the target ACOX1 protein. A redocking validation step was performed illustrated in Figure 1a for PPAR-α and Figure 1b ACOX-1. Quinoline-Thiazolidine-2, 4-Dione **8** showed a binding energy of −8.02 kcal/mol with PPARα (PDB: 2ZNN) and an RMSD of 1.38 Å. The compound formed hydrogen bonds with HIS440 and TYR464, residues that are important for stabilizing ligands in the PPARα pocket (Figure 2). For ACOX1 (PDB: 7Q86), the binding energy for the Quinoline-Thiazolidine-2, 4-Dione **8** was −7.73 kcal/mol with an RMSD of 0.68 Å. The ligand fit into the canonical binding site and formed a hydrogen bond with GLN138 (DHA angle 156.7°). Hydrophobic contacts with TYR232 and PHE420 further stabilized the compound in the ACOX1 pocket (Figure 3). Table 1 summarizes the docking scores, RMSD values, and interaction residues for both proteins.

### 2.3. In Vivo Effects of Hybrid on Zebrafish

**Figure 4:**
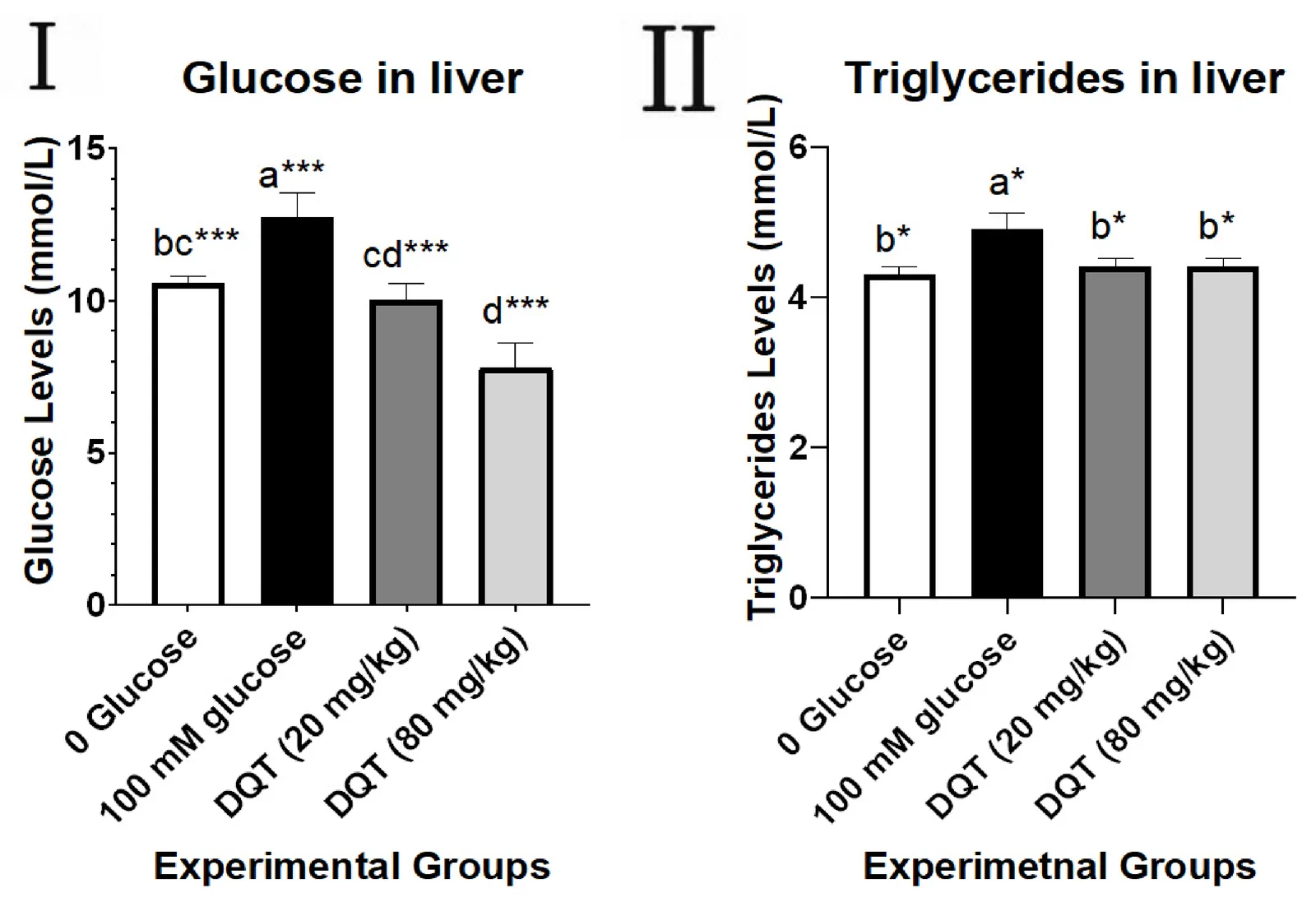
Levels (mmol L^−1^) of glucose (**I**) and triglycerides (**II**) in zebrafish livers in the experimental groups: 0 mM glucose (negative control), 100 mM glucose (positive control), and 100 mM glucose-then-quinoline-thiazolidine-2,4-dione (DQT) oral administration (20 and 80 mg/kg fish weight). Results are expressed as mean ± standard error of the mean (SEM). Different black letters above the bars of each experimental group indicate significant differences between them. Triple asterisk (***): *p* < 0.001, single asterisk (*): *p* < 0.05.

The positive control group, which had persistent glucose exposure before the recovery period, exhibited significant elevations in both glucose and triglyceride levels compared to all other groups (Figure 4I,II). Triglyceride levels in the negative control group were approximately 1.5–2 folds lower than those in the positive control group. The groups treated with low and high doses of Quinoline-Thiazolidine-2, 4-Dione (DQT) showed significant reductions in glucose and triglyceride levels, comparable to those in the negative control group (Figure 4I,II). For glucose, the group treated with a high DQT dose showed slightly lower but still significant levels compared to those in the negative control group (Figure 4I).

**Figure 5:**
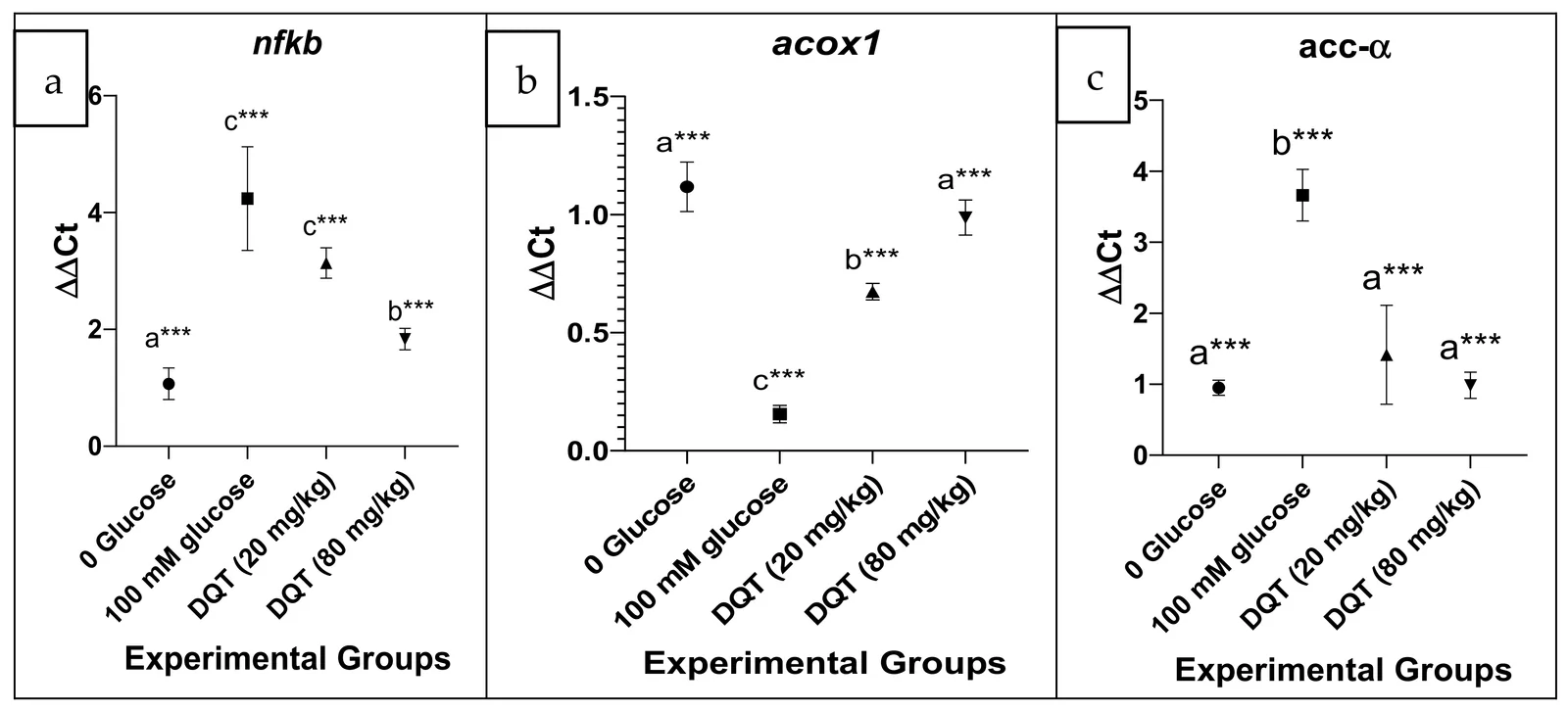
(**a**–**c**) mRNA expression levels (∆∆Ct) of; (**a**) nuclear factor kappa beta (*NF-κB*), (**b**) peroxisomal acyl-coenzyme A oxidase 1 (*acox1*); (**c**) acetyl-CoA carboxylase α (*accα*), in zebrafish liver homogenates in the following experimental groups: 0 mM glucose (negative control), 100 mM glucose (positive control), 100 mM glucose-then-20 mg/kg fish weight of DQT, and 100 mM glucose-then-80 mg/kg fish weight of DQT (orally administered). The results are expressed as the mean ± standard error of the mean (SEM). Different black letters above the bars of each experimental group indicate significant differences among them. Triple asterisk (***): *p* < 0.001.

The expression analysis revealed that hyperglycemia markedly altered inflammatory and metabolic gene regulation, which was effectively modulated by DQT treatment. The key inflammatory mediator related to T2DM development is *nf-κβ*. mRNA expression levels were significantly elevated in the positive control group compared to all other groups, whereas high-dose DQT administration markedly reduced its expression, approaching near-normal levels, (Figure 5a). However, the lipolytic enzyme transcript *acox1* was strongly suppressed under hyperglycemic conditions but showed partial restoration with low-dose DQT and complete normalization with high-dose treatment, (Figure 5b). In addition, the lipogenic enzyme transcript *accα* exhibited several-fold upregulation in the positive control group relative to the negative control. Both low- and high-dose DQT treatments significantly downregulated *accα*, restoring its expression to baseline levels comparable to the negative control, (Figure 5c).

## 3. Discussion

Peroxisome proliferator-activated receptor alpha (PPAR-α) and acyl-CoA oxidase 1 (ACOX-1) are two key regulators of lipid metabolism and fatty acid β-oxidation, processes that are often disrupted in type 2 diabetes. PPARα controls the expression of genes involved in lipid transport and oxidation, while ACOX1 carries out the first step of peroxisomal β-oxidation. Problems in these pathways contribute to insulin resistance, which makes both proteins important therapeutic targets.

In this context, the synthesized Quinoline-Thiazolidine-2, 4-Dione hybrid **8** was docked into the ligand-binding cavities of PPAR-α and ACOX-1. The interaction profile upon docking in PPAR-α revealed that the compound engages key amino acid residues commonly involved in the binding of the co-crystallized ligand, notably HIS440, which plays a role in stabilizing ligands within the PPAR-α binding pocket. In the docking of Quinoline-Thiazolidine-2, 4-Dione **8** into the second target (ACOX-1), the ligand occupied the binding site, and the interaction analysis revealed three key bonds, including a hydrogen bond between GLN138 and the oxothiazolidine ring, with a DHA angle of 156.7° supporting a stable and directional interaction. Additionally, two hydrophobic contacts with Thr232 and Phe420 further contributed to ligand stabilization within the ACOX-1 pocket. The binding energies (−8.02 and −7.73 kcal/mol) are close in value, which supports the idea that hybrid 8 can act as a dual modulator. These findings point to hybrid **8** as a promising starting scaffold for developing multitarget agents.

Furthermore, the capabilities of the new Acetamidoquinoline analog in preventing the development of hyperglycemia and hypertriglyceridemia could be elucidated by using zebrafish (Danio rerio) as a model. This is particularly important for various reasons. First, there is a clear and well-established relationship between these two symptoms and the development of diabetes mellitus type II. Second, it contributes more knowledge about the success of zebrafish as a vertebrate model for studying these metabolic disorders and the new compounds that aim to reduce them.

In the current study, the positive control group showed upregulation of the key inflammatory mediator NF-κB and the lipogenic enzyme transcript *Accα.* This was accompanied by a significant increase in glucose and triglyceride levels. Furthermore, the same group exhibited reduced insulin levels and the transcription of the primary lipolytic enzyme gene, *Acox1.* All these changes are interrelated. Persistent hyperglycemia activates *NF-κB*, which subsequently leads to the activation of different cytokines and inflammatory mediators that eventually lead to increased systematic inflammation and development of insulin resistance, among many other related symptoms [22,23]. Alterations in the regulatory role of insulin during chronic inflammatory status related to T2DM alter the insulin-mediated modulation of lipoprotein lipase (LPL), which stimulates *de novo* TG synthesis [21]. Moreover, persistent hyperglycemia increases the accumulation of misfolded proinsulin in β islets of Langerhans, overproduction of reactive oxygen species, enhancement of proapotoic signals, and finally proinsulin mRNA degradation and local islets inflammation [7,24]. Lipotoxicity, which is common in obesity, induces metabolic and oxidative stress that eventually leads to β islets damage and defective insulin production [24]. Moreover, hyperglycemia is strongly related to hypertriglyceridemia (HTG) [25]. The development of insulin resistance is also directly related to obesity, which is currently accepted to be one of the leading risk factors for T2DM. HTG is related to TG-rich lipoprotein overproduction due to increased release of free fatty acids from adipose tissue and enhanced hepatic lipogenesis [26].

Therefore, the pattern of increase in both glucose and TG found in zebrafish chronically subjected to 100 mM glucose in the current study, together with a decrease in insulin levels, can indicate an inflammatory state in the β-islets of Langerhans. Furthermore, the upregulation of the *de novo* lipogenic enzyme transcript, i.e., Accα, can further support the presence of status of hepatic lipotoxicity, probably related to TG accumulation, as described before for these enzymes in response to this scenario of liver lipotoxicity [27].

However, the application of Quinoline-Thiazolidine-2, 4-Dione in the current study was related to alleviating the effect in fish groups that were previously stressed with prolonged glucose exposure, which led to persistent hyperglycemia, even after the transfer of fish to glucose-free water. Both doses of Quinoline-Thiazolidine-2, 4-Dione restored the insulin, glucose, TG, *nf-κβ*, *Acox1*, and *accα* levels to normal, that is, the levels in the negative control group. This directly refers to the capability of this drug to treat the adverse effects of hyperglycemia and hypertriglyceridemia in the tested vertebrate model. Recently, quinolines have been elucidated as successful therapeutic agents against nonalcoholic fatty liver disease and T2DM [28,29,30]. Their roles in T2DM mainly include competitive inhibition of α-glucosidase [31]. Inhibition of this enzyme leads to a reduction in the bioavailability of glucose for intestinal absorption [31,32]. To the authors’ knowledge, few studies have assessed the interaction between quinoline derivatives and lipogenic/lipolytic enzymes. However, some studies have shown the potential capabilities of these derivatives in reducing inflammatory reactions and enhancing lipid catabolism. Of these, Berberine (BBR), a quinoline alkaloid, was found to be very effective in reducing fatty acid synthesis and accumulation through various mechanisms, including upregulation of lipolytic enzyme systems, such as the activation of PPAR-α and ACOX1 expression [33,34]. This directly leads to a reduction in hepatic steatosis and its negative impacts on normal liver functionality [30].

Finally, another possibility that cannot be ruled out for the ameliorative effect of the applied Quinoline-Thiazolidine-2, 4-Dione is the previously elucidated stimulatory effect of quinoline derivatives on pancreatic β-islets regeneration and long-term insulin secretion enhancement [35]. This capability of quinoline derivatives emerges from their roles as potential inhibitors of dual-specificity tyrosine phosphorylation-regulated kinase A1 (DYRKA1) [36,37]. Hence, damage to the β-islets of Langerhans that could occur in glucose-challenged zebrafish could be alleviated by the applied quinoline analog, which can explain the restoration of insulin, glucose, and TG levels to the normal control after treatment with this quinoline derivative.

Considering the above-mentioned information, the applied Quinoline-Thiazolidine-2, 4-Dione showed promising effects in treating zebrafish with persistent hyperglycemia and hypertriglyceridemia. This drug could restore several parameters related to lipotoxicity and hyperglycemia to their normal state.

## 4. Materials and Methods

### 4.1. Characterization Techniques and Chemical Reagents

Fourier transform infrared (FTIR) spectra were recorded at the Applied Nucleic Acid Research Center, Faculty of Science, Zagazig University, Egypt, using an attenuated total reflectance (ATR) technique over the wavenumber range of 4000–400 cm^−1^. The characteristic absorption bands are reported as ν_max in cm^−1^.The ^1^H and ^13^C NMR spectra (*δ*, ppm) were performed on DELTA2 NMR (500 and 125 MHz) and BRUKER Avance III (400 and 100 MHz) spectrometers at National Research Center, Cairo, Egypt, using tetramethyl silane (TMS) as an internal standard and deuterated dimethyl sulfoxide (DMSO-*d*_6_) as a solvent (Thermo Fisher DIONEX, Bavaria, Germany). Mass spectra were recorded at the National Center for Fungi, Al-Azhar University, Cairo, Egypt, using a GC–MS system equipped with electron ionization (EI) source operated at 70 eV. The molecular ion and major fragment ions are reported as *m*/*z* values. The purity of the synthesized compounds was assessed by HPLC at the Nuclear Materials Authority, Cairo, Egypt, using a Thermo Scientific Dionex UltiMate 3000 HPLC system equipped with a diode-array detector (DAD). Chromatographic analysis was performed at 254 nm using a reversed-phase C8 column (4.6 mm i.d., 5 μm particle size) with an injection volume of 20 μL. An isocratic mobile phase of acetonitrile/water (70:30, *v*/*v*) was used. The purity of compound 8 was determined based on the integrated chromatographic peak area. Commercially available starting materials, including 4,7-dichloroquinoline (99.0%), 1,3-diaminopropane (99.0%), maleic anhydride (98.0%), thiourea (98.0%), dimethylformamide (DMF), concentrated hydrochloric acid (HCl), thionyl chloride, and triethylamine (TEA), were purchased from Sigma-Aldrich, Cairo, Egypt, and used as received without further purification. Hexane, 1,4-dioxane, and ethanol were purchased from Al-Gomhoria Company, Cairo, Egypt and used without further purification. Reaction progress and purity were monitored by thin-layer chromatography (TLC) on precoated silica gel 60 F254 plates (Merck, Darmstadt, Germany), with visualization under UV light at 254 nm. Intermediates 2-(2,4-dioxothiazolidin-5-yl)acetyl chloride **4** [20] and N^1^-(7-chloro quinolin-4-yl) propane-1,3-diamine **7** [38] were synthesized according to previously reported procedures. Structure characterization (^1^H-NMR, ^13^C-NMR, EI-MS) and compound purity results (HPLC) are illustrated in Supplementary Material File S1.

### 4.2. Proceduers and Characterization of the Synthesized Compounds 

#### 4.2.1. Synthesis of 2,4-Dioxothiazolidine-5-acetic Acid **3**

A mixture of 19.6 g (0.2 mol) of maleic anhydride **1** and 15.2 g (0.2 mol) of thiourea (**2**) in 50 mL of concentrated hydrochloric acid was boiled for 5 h and cooled. The precipitate was separated by filtration, washed with water, dried, and recrystallized from water to obtain pure compound **3** (yield = 72%; m.p. 166–169 °C). Reported in [20]: m.p., 168 °C.

#### 4.2.2. Synthesis of 2,4-Dioxothiazolidine-5-acetyl Chloride **4**

A mixture of 8.75 g (0.05 mol) of acid **3** and 11.9 g of thionyl chloride in 30 mL of dioxane was heated for 5 h, cooled, and treated with 100 mL of hexane. The precipitate was separated by filtration and recrystallized from toluene to obtain compound **4** in a yield of 70%; its melting point was 118–120 °C. Reported in [20]: m.p., 119 °C.

#### 4.2.3. Synthesis of the Intermediate N^1^-(7-chloroquinolin-4-yl) propane-1,3-diamine (**7**)

To 4,7-dichloroquinoline **5** (1.0 equiv), 1, 3-diaminopropane **6** (12.0 equiv) was added. The reaction was performed neat at reflux for 2 h. The excess propane-1,3-diamine evaporated under heat and vacuum. The remaining waxy solid was suspended in 200 mL of water and stirred for 20 min. The solid suspension was filtered to obtain a pure white product. Yield (89%), m.p. 118–121 °C. Reported in [21]: m.p.,120 °C. FTIR (KBr, cm^−1^): 2714, 2882, 3109 (C-H, stretch), 3398, 3669, 3824 (N-H, stretch), 1374 (C-N). ^1^H NMR (400 MHz, CDCl_3_) δ ppm:6.30–8.8 (m, 5H, Ar-H), 8.35 (s, 1H, NH), 7.28 (s, 2H, NH), 3.30 (m, 2H, CH_2_), 3.20 (m, 2H, CH_2_), 1.80 (m, 2H, CH_2_). ^13^C NMR (400 MHz, CDCl_3_) δ ppm: 143, 139, 128, 127, 125, 124, 122, 118, 98 (Aromatic carbon), 48, 34, 34 (Aliphatic carbon).

#### 4.2.4. Synthesis of N-(3-((7-chloroquinolin-4-yl)amino)propyl)-2-(2,4-dioxothiazolidin-5-yl)acetamide (Quinoline-thiazolidine-2,4-dione hybrid) **8**

A mixture of an equivalent amount of 2,4-dioxothiazolidine-5-acetyl chloride **4** and N^1^-(7-chloroquinolin-4-yl)-propane-1,3-diamine **7** was dissolved in dry DMF in the presence of triethylamine (3 eq), the reaction mixture was refluxed with stirring, and the reaction progress was monitored by TLC for 6 h. The mixture was then poured onto crushed ice, and the resulting precipitate was collected by suction filtration to obtain the crude products, which were recrystallized using ethanol.

Racemic mixture obtained with a yield of 53%; light brown solid; m.p: >300 °C, (Yield 53%); light brown solid; m.p: >300 °C, FTIR (ATR, ν_max, cm^−1^): 3410 (N–H), 3245 (amide N–H), 1735 (C=O), 1685 (C=O), 1590 (C=C), 1535 (C=N), 725 (C–Cl). ^1^H NMR (600 MHz, DMSO-d_6_, δ ppm): 10.22 (s, 1H), 9.10 (s, 1H), 8.50 (s, 1H), 8.10 (d, J = 5.4 Hz, 1H), 7.75 (dd, J = 9.0, 2.5 Hz, 1H), 7.60 (d, J = 5.4 Hz, 1H), 7.50 (d, J = 9.0 Hz, 1H), 4.65 (dd, J = 10.2, 4.0 Hz, 1H), 3.40–3.30 (m, 2H), 3.10 (dd, J = 15.8, 10.2 Hz, 1H), 2.90 (dd, J = 15.8, 4.0 Hz, 1H), 1.85 (quint, J = 6.8 Hz, 2H). ^13^C NMR (125 MHz, DMSO-d_6_, δ ppm): 196.0, 171.2, 167.5, 152.4, 149.3, 141.8, 135.9, 129.2, 129.1, 128.9, 128.7, 126.6, 124.8, 122.6, 55.2, 42.0, 29.5. MS (EI): *m*/*z* 392.23 [M] +; calcd. for C_17_H_17_ClN_4_O_3_S: 392.07. HPLC purity: 95%.

### 4.3. Proceduers for Docking Study

The target protein was downloaded from the Protein Data Bank and complexed with its co-crystallized ligand, with a PDB ID of 2ZNN and 7Q86 for PPAR-1 and acox-1 respectively. Compound structure was sketched as 2d structure in chem-sketch then converted to 3d using open babel and saved in sdf format, which can be opened in UCSF-chimera version 1.17.3 and Discovery Studio. Ligands and proteins were then prepared using UCSF-Chimera by adding hydrogen and assigning Gasteiger charges, followed by energy minimization. Binding site is defined by the cocrystallized ligand with axis for PPAR-1 x = 11.01, y = 4.99, z = −8.29, and for acox-1 x = 14.94, y = 16.54, z = 23.40. Docking was performed using Vina in chimera 1.17.3. The results were saved as PDBQT and subsequently visualized using Biovia Discovery Studio v21.1.0.20298 [39].

### 4.4. In Vivo Testing of the Target Hybrid

**Laboratory acclimation of experimental fish**

Zebrafish Danio rerio mixed-sex adults were obtained from commercial pet shops and maintained at normal water temperatures (22 ± 2 °C) and a light: dark cycle (12 h: 12 h) for two weeks before starting the experiment. These conditions were adopted from the previously established experimental protocol used in our study. The same environmental conditions were maintained consistently throughout the experimental period to minimize variability among the experimental groups. The fish were fed once a day with a diet for tropical fish aquaria, encompassing 23% crude protein, 3% crude fat,1.5% crude fiber, 8.0% 0.7% moisture, and phosphorus. The feeding ratio was 2% of total body mass daily. The fish were maintained in 14 L plastic aquaria equipped with mechanical and biological filters. To maintain water quality, 50% of the water was replaced daily following the cleaning of the tank to remove waste [40]. The tanks were monitored daily to record any instances of mortality.

### 4.5. In Vivo Drugs Testing

#### 4.5.1. Experimental Procedures

All experimental procedures were performed in accordance with the guidelines approved by the Institutional Animal Care and Use Committee (IACUC) of the Department of Zoology in the Faculty of Science of Menoufia University, Egypt (approval number MUFSFGE225, DATE 4 February 2025). Feeding continued until the end of the experiment and was stopped one day before a transfer or sampling event. A group of zebrafish (n = 60, weight: 1.8 ± 0.4 g, length: 1.5 ± 0.5 cm) was placed in a 14 L aquarium containing 100 mM D-glucose (dextrose monohydrate, MF: C_6_H_12_O_6_.H_2_O, Cat. No. G002, Piochem, Egypt) solution in water. Another group (n = 15) was maintained until the end of the experiment in a 4 L aquarium without glucose to serve as a negative control. Both groups were kept in their aquaria for 12 days. On day 13, Thirty zebrafish were randomly distributed into three new 4 L aquaria (n = 10 zebrafish/aquarium) containing glucose-free, dechlorinated tap water and were maintained there for an additional day, with appropriate group-specific labeling for each aquarium. An additional thirty zebrafish were kept in the same aquaria under identical experimental conditions, solely for mortality monitoring, and were excluded from all experimental analyses. The groups originating from the initial glucose immersion tank were fed orally with either fish feed alone or fish feed mixed with the drug. Consequently, three groups were delineated: the first received a normal diet without any drug, serving as a potential hyperglycemia positive control. The second and third groups received 20 and 80 mg/kg fish weight of Quinoline-Thiazolidine-2, 4-Dione. The doses were selected as the averages among different studies that confirmed the anti-inflammatory and antidiabetic effects of thiazolidinidione analogues [41].

Feeding continued for four days. Thereafter, the fish were euthanized by placing them in ice-cold water (2–4 °C). Each fish was dissected, and the entire livers were separately collected and preserved as described by Galal-Khallaf et al. [42]. The livers of five fish were removed and placed in 10 (*w*/*v*) of RNAlater (Invitrogen™ AM7021) at 4 °C overnight, to be transferred to − 20 °C the next day, where they were kept until subsequent gene expression. The livers of the remaining five fish in each experimental group were placed in sterile 1.5 mL tubes containing 500 µL of ice-cold phosphate buffer (KH_2_PO_4_, pH = 7.2, 10 mM). These tubes were separately and immediately homogenized. The homogenates were then centrifuged at 10,000g for 10 min at 4 °C. The supernatants were transferred to new sterile 1.5 mL tubes. These new tubes were stored at − 80 °C until measuring glucose, triglyceride, and insulin concentrations.

#### 4.5.2. Assessment of Energetic Metabolites Levels

Glucose levels were quantified using a commercial kit (Spinreact, cat. No. 1001191), while triglyceride levels were assessed using another commercial kit from Spinreact (Cat. no. 1001310). Measurement of both energetic metabolites in zebrafish tissues followed the method that is commonly applied for different fish species, including zebrafish [4,43,44]. In both cases, KH_2_PO_4_ (10 mM, pH 7) was used as a blank. Homogenates of hyperglycemia-induced and control zebrafish were applied in triplicate in a 96-well microplate for each treatment. Intra-specific errors were calculated as the standard error of the mean of the measurements between the triplicate samples. After adding the reagents to the sample, negative control, blank, and standard curve wells, the plates were read at 500 nM in a spectrophotometric plate reader (Tecan, infinite F50, Mannedorf, Switzerland).

#### 4.5.3. RNA Extraction and QPCR

After removal from RNAlater™, total RNA was extracted using the GeneJET RNA Purification Kit (Thermo Scientific, Waltham, MA, USA; Cat. No. K0731) without modifications to the tissue-extraction protocol. RNA quantity and purity were quantified using spectrophotometric measurements at 260 nm and 260/280 nm, respectively. RNA quality was checked using ethidium bromide-stained RNase-free 1% agarose gel electrophoresis. With the best quality, purity (1.8–2), and quantity of RNA, 500 ng of total RNA was subjected to reverse transcription using the ABT-H minus cDNA synthesis kit (Applied Biotechnology, Ismailia, Egypt), following the manufacturer’s protocol. Subsequently, quantitative polymerase chain reaction (QPCR) was performed using 2 × ABT SYBR Mix with low ROX (Applied Biotechnology, Egypt), UltraFlux^®^flat 0.2 mL flat cap PCR strips (Cat. No. 3135-00, SSIbio, Lodi, CA, USA), and Applied Biosystems 7300 Real-Time PCR System. The targeted transcripts for QPCR amplification in the current study included the key inflammatory mediator related to T2DM development, namely, nuclear factor kappa beta (nf-κβ); de novo lipogenesis enzyme transcript [i.e., acetyl-CoA carboxylase alpha (accα)]; and the β-oxidation primary enzyme gene responsible for lipid catabolism, namely, acetyl-CoA oxidase 1 (acox1). As an internal reference, beta-actin (actb) was measured in each sample based on its very low Ct differences among different samples. The sequences of all QPCR primers applied in the current study, in addition to the expected amplicon sizes and applied QPCR program, are listed in Table 2. Finally, a dissociation curve was generated for all PCRs to confirm the existence of a single amplicon for each transcript. The 2^−ΔΔCt^ method was used to calculate the expression of target transcripts.

### 4.6. Data Analysis

Statistical analyses for significance between groups in glucose, insulin, and reference-gene-normalized results for mRNA expression levels of target transcripts were analyzed using one-way analysis of variance (ANOVA). Differences were considered significant at *p* < 0.01. Least significant difference (LSD) was used as a post hoc test for accurate differentiation of significant groups for each parameter assessed. All statistical analyses were performed using Statgraphics Centurion XVI, version 16.2.04 (StatPoint Technologies, Inc., Warrenton, VA, USA).

## 5. Conclusions

In this study, we successfully designed and synthesized a novel Quinoline-Thiazolidine-2, 4-Dione hybrid that demonstrated significant antihyperglycemic and antihyperlipidemic activities. Structural characterization confirmed the integrity of the synthesized compound, while molecular docking revealed strong binding affinities toward PPAR-α and ACOX-1, supporting the compound’s potential role in regulating lipid metabolism and glucose homeostasis. In vivo evaluation using zebrafish models further validated its efficacy, as treatment restored glucose, triglyceride, and insulin levels and normalized the expression of key metabolic and inflammatory genes, including NF-κB, ACOX1, and ACCα. Notably, the compound exhibited dose-dependent improvements without apparent toxicity, underscoring its therapeutic promise. Collectively, these findings highlight the hybrid scaffold as a promising lead for the management of type 2 diabetes mellitus and associated dyslipidemia. Further studies on the applied drug should be conducted to confirm its efficacy against T2DM in higher vertebrate models.

## Figures and Tables

**Table 1:** Docking results for Quinoline-Thiazolidine-2, 4-Dione **8** in the binding pocket of PPAR-α and *ACOX-1*, (PDB ID 2ZNN and7Q86 respectively).

Target PDB ID	Binding Scores (kcal/mol)	RMSD (Å)	Residues of Interaction	Type of Interaction
2ZNN	−8.02	1.38	HIS 440 TYR 464	H-bond (H-bond donor) H-bond (H-bond donor)
7Q86	−7.73	0.68	GLN 138 PHE 420 TYR 232	H-bond (H-bond donor) Hydrophobic (π-π-stacking) Hydrophobic (π-π-T-shaped)

**Table 2:** Gene names, gene bank locus, primer sequence, and amplicon length of the applied qPCR primers.

Gene	Locus ID	Amplic on Size (bp)	Forward Primer (5′ → 3′)	Reverse Primer (5′ → 3′)
*FASN*	XM_682295	250	ATCTGTTCCTGTTCGATGGC	AGCATATCTCGGCTGACGTT
*ACOX1*	BC097101.1	162	GCACGGATGTGTGTACCGTGC	GCGTCCAGAGCCCCTTGACCT
*ACC-α*	NM_001271308	194	AGAGAGGGCAGGTTTTACCA	GCCATCATACGAGAGCAACA
*NF-κB*	NM_001003414	202	GGTCGGACAGAGATCACGGATT	TGCTGTTCTTCACGTCCTCT

## Data Availability

The original contributions presented in this study are included in the article/Supplementary Material. Further inquiries can be directed to the corresponding author.

## Supplementary Materials

The following supporting information can be downloaded at: https://www.mdpi.com/article/10.3390/ph19091490/s1, Supplementary Material File S1: structure characterization (^1^H-NMR, ^13^C-NMR, EI-MS) and compound purity results (HPLC).
