# Supplementary material for: Design, Synthesis and Antihyperglycemic/Antilipidemic Activities of Quinoline-thiazolidine-2,4-dione Hybrid
Source: Pharmaceuticals (Basel). 2026 Sep 19;19(9):1490. doi: 10.3390/ph19091490 (PMC13610976; doi:10.3390/ph19091490)
Supplement: Supplementary file 1 [file pharmaceuticals-19-01490-s001.zip › pharmaceuticals-4520687-supplementary.pdf]

Article

Design, Synthesis and Antihyperglycemic/Antilipidemic Activities of Quinoline-thiazolidine-2,4-dione Hybrid

ElShimaa Ahmed Mohamed Abousaada [1], Asmaa Galal-Khallaf [2], Khaled Mohammed-Geba [2], Mohamed El-Bahnsawye [1], Mona K. Abo Hussein [3], Elshaymaa I. Elmongy [4,*], Rania Ali Ahmed Shaltout [2], Reem Binsuwaidan [5], Hadeer Ali [1], Ahmed I. El-Tantawy [6], Hamed Abdel-Bary [1] and Ibrahim El Tantawy El Sayed [1]

1 Chemistry Department, Faculty of Science, Menoufia University, Shebin El-Kom 32511, Egypt; alshaimaaahmed95@gmail.com (E.A.M.A.); m_nh22010@yahoo.com (M.E.-B.); yohader1@gmail.com (H.A.); hamedabdelbary2006@yahoo.com (H.A.-B.); ibrahimtantawy@science.menofia.edu.eg (I.E.T.E.S.)

2 Zoology Department, Molecular Biology and Biotechnology Laboratory, Faculty of Science, Menoufia University, Shebin El-Kom 32511, Egypt; as_kh_22@yahoo.com (A.G.-K.); khaledmohammed@science.menofia.edu.eg (K.M.-G.); raniashaltout606@gmail.com (R.A.A.S.)

3 Clinical Microbiology and Immunology Department, National Liver Institute, Menoufia University, Shebin El-Kom 32511, Egypt; monaabohussein@yahoo.com

4 Pharmaceutical Chemistry Department, Faculty of Pharmacy, Capital University (Formerly Helwan University), Ain Helwan, Cairo 11795, Egypt

5 Department of Pharmaceutical Sciences, College of Pharmacy, Princess Nourah Bint Abdulrahman University, P.O. Box 84428, Riyadh 11671, Saudi Arabia; rabinsuwaidan@pnu.edu.sa

6 Research Institute for Interdisciplinary Science, Okayama University, Tsushima-naka, Kita-ku, Okayama 700-8530, Japan; chemahmed293@gmail.com

* Correspondence: shaymaa.taha@pharm.capu.edu.eg

Supplementary Material File S1: structure characterization (1H-NMR, 13C-NMR, EI-MS) and compound purity results (HPLC).

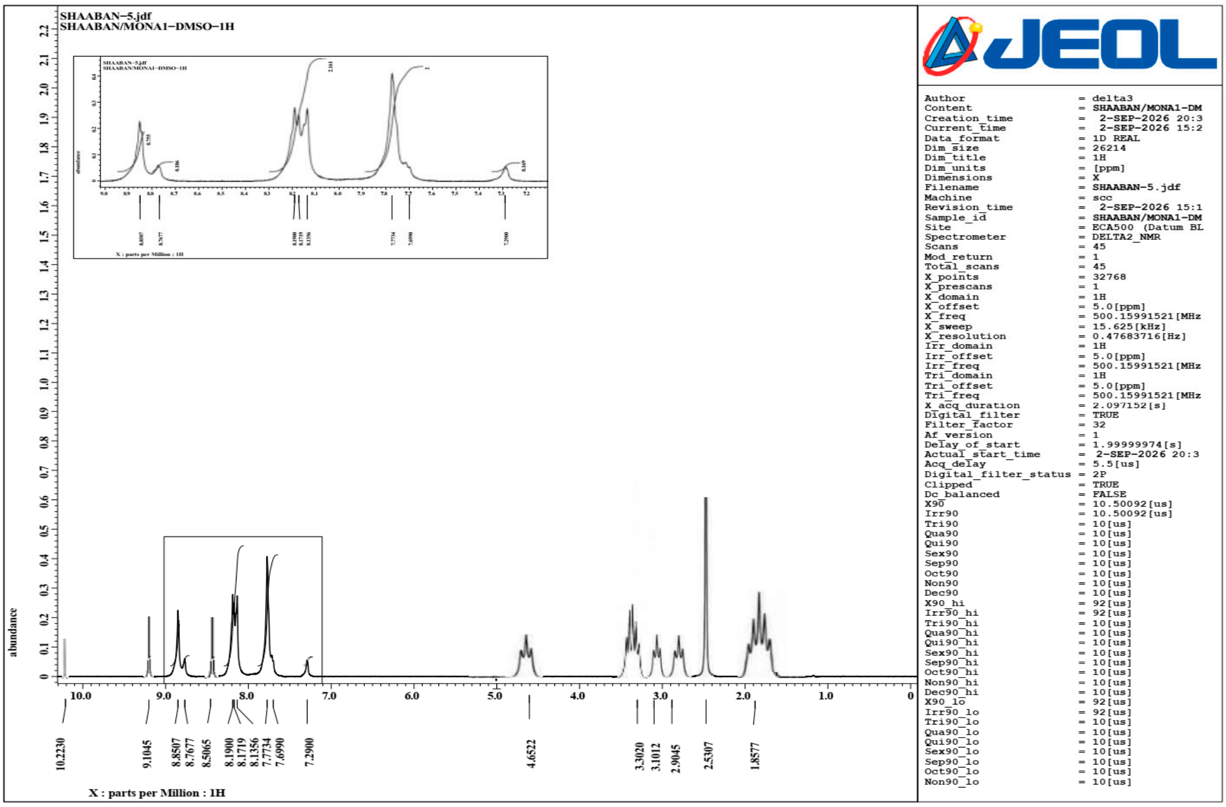

^{1}H NMR of hybrid 8

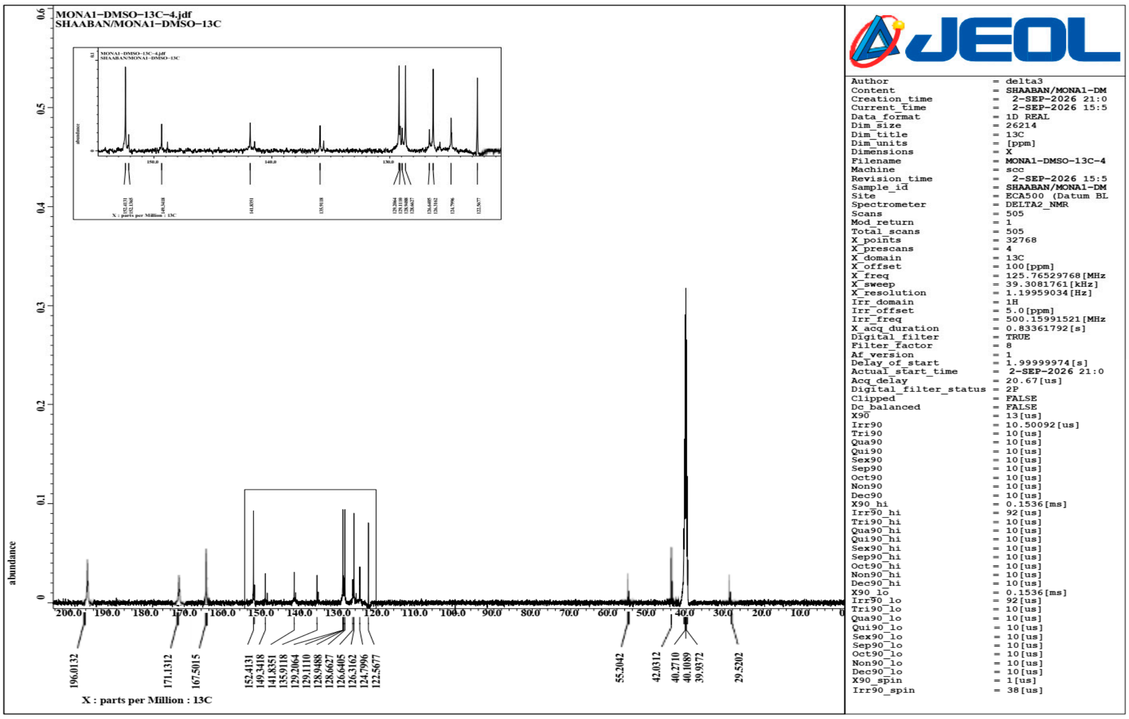

^{13}C NMR of hybrid 8

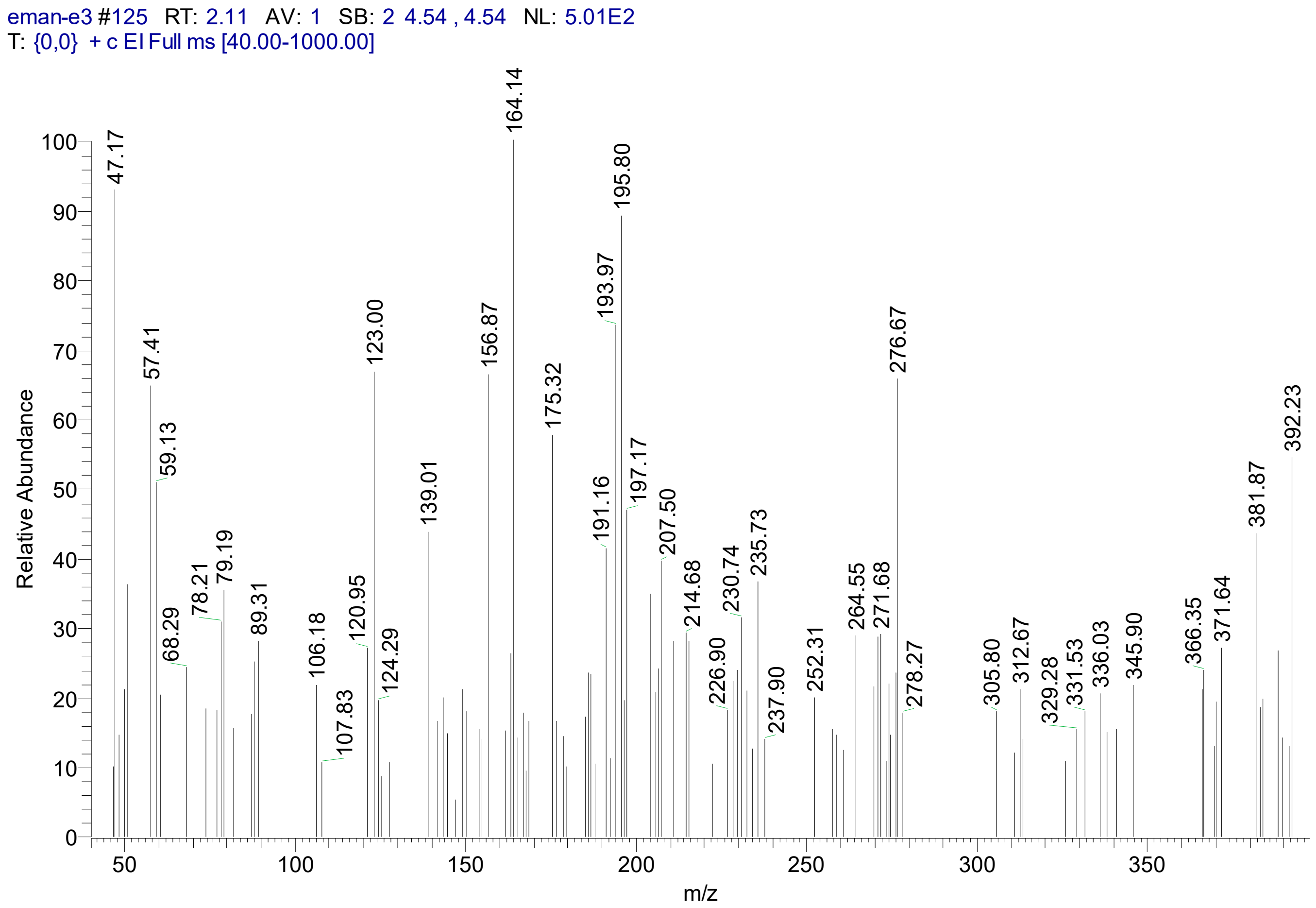

EI-MS fragmentation of hybrid 8

HPLC for hybrid 8:

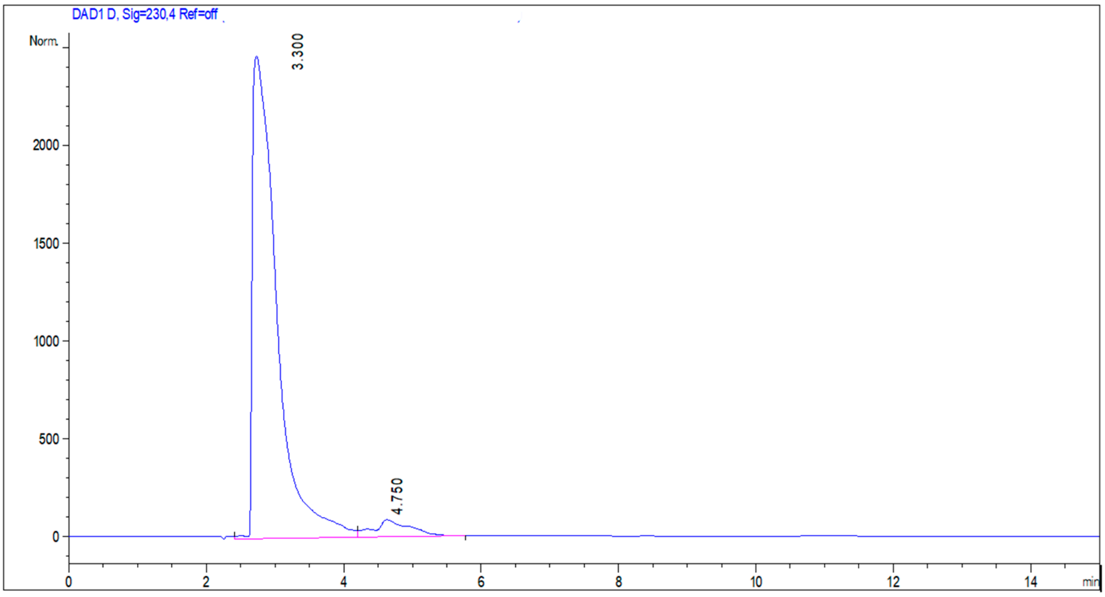

#	Time	Area	Height	Width	Area%	Symmetry
1	3.3	60588.8	2463.7	0.4099	95.106	10.266
2	4.75	3117.7	86.2	0.603	4.894	1.254

HPLC method and conditions:

Agilent 1100 liquid chromatography integrated system equipped with G1313A automated injector, G1311A quaternary pump and G1315B diode-array detector (DAD) operated at a wavelength of 260 and 330 nm. The chromatographic separation of the compounds was achieved with a reversed phase column, ZORBAX XDB-C18 (5.0 x 250, 5 µm)) from Agilent (USA) operating at constant temperature (30oC). 10 mg samples were dissolved in 10 mL of Eluent consist of water: methanol (50:50), filtered through a 0.45 µm nylon filter and injected into the HPLC. The samples were eluted at a flow rate of 1 mL/minute and linear gradient started by 10% water and 90 % methanol to 90% water and 10% methanol after 20 min.
